# Supplementary material for: In-vivo programmable acoustic manipulation of genetically engineered bacteria
Source: Nat Commun. 2023 Jun 6;14:3297. doi: 10.1038/s41467-023-38814-w (PMC10244463; doi:10.1038/s41467-023-38814-w)
Supplement: Supplementary file 1 — Supplementary Information [file 41467_2023_38814_MOESM1_ESM.pdf]

Supplementary Information

In-vivo programmable acoustic manipulation of genetically engineered bacteria

Ye Yang[1,2], Yaozhang Yang[1,3], Dingyuan Liu[1], Yuanyuan Wang[1], Minqiao Lu[1], Qi Zhang[1], Jiqing Huang[1], Yongchuan Li[1], Teng Ma[1,2], Fei Yan[1,2], Hairong Zheng[1,2].

[1] *Shenzhen Institutes of Advanced Technology, Chinese Academy of Sciences, Shenzhen 518055, China.*

[2] *Shenzhen College of Advanced Technology, University of the Chinese Academy of Sciences, Beijing 100049, China.*

[3] *Shenzhen Bay Laboratory, Shenzhen 518132, China.*

These authors contributed equally: Ye Yang, Yaozhang Yang.

Correspondence should be addressed to T.M. (E-mail: teng.ma@siat.ac.cn); F.Y. (Email: fei.yan@siat.ac.cn); H.Z. (Email: hr.zheng@siat.ac.cn).

Supplementary Figures

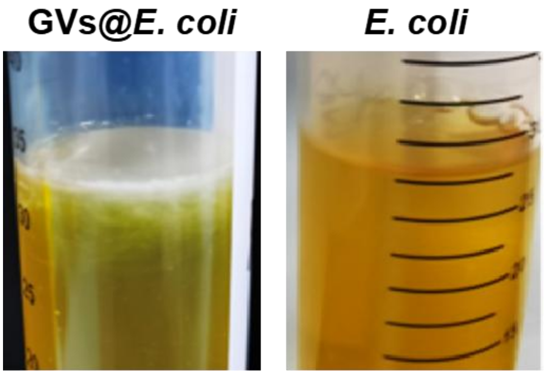

Supplementary Fig. 1 | Isolation of GVs@*E. coli*. The genetically engineered GVs@*E. coli* float up after centrifugation at 300 g for 3 h, and white floating bacteria appear in the upper layer. By contrast, no bacteria float in the uninduced control *E. coli* group.

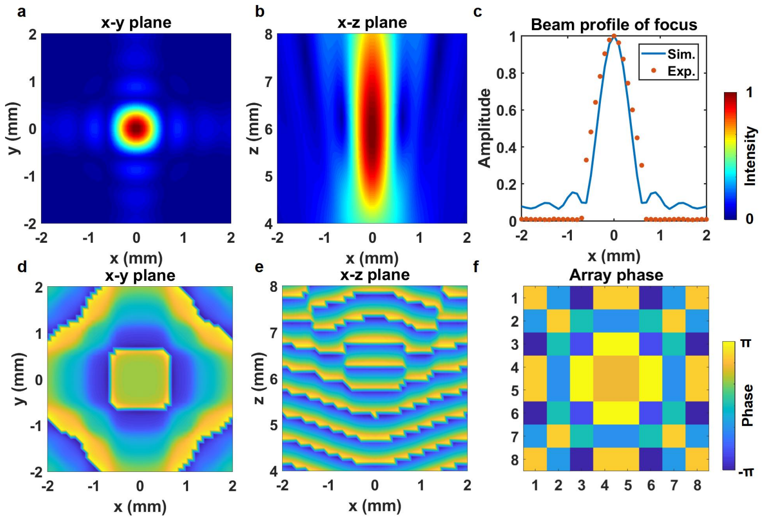

Supplementary Fig. 2 | Focal acoustic field (focal point: (0, 0, 6) mm). a Intensity of the acoustic field in the x-y plane (focal depth = 6 mm). **b** Intensity of the acoustic field in the x-z plane (y = 0). **c** Simulated and experimental beam profiles of the field (y = 0 and focal depth = 6 mm). **d** Phase of the acoustic field in the x-y plane (focal depth = 6 mm). **e** Phase of the acoustic field in the x-z plane (y = 0). **f** Phases of the element in the array for generating the focal field.

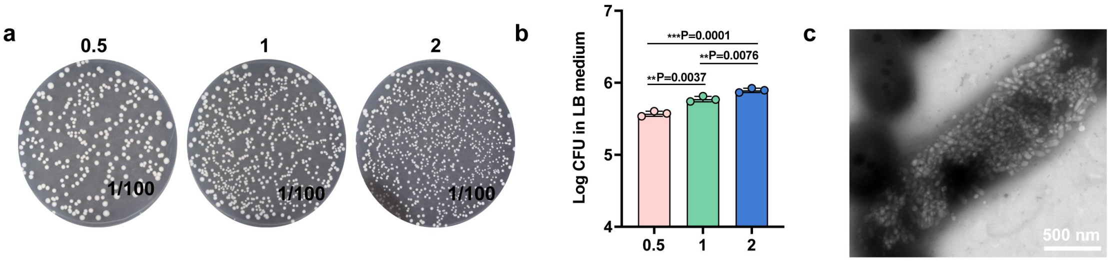

Supplementary Fig. 3 | The number of GVs@*E.coli* in a typical cluster a The number of bacteria in the cluster, when ultrasound effected on different concentrations of bacteria (OD_{600nm} =0.5, 1, 2). **b** Quantification of **a**, n = 3 biologically independent samples per group. Data were presented as mean ± s.d. **c** Transmission electron microscope image of GVs@*E.coli* after ultrasound irradiation. Images were representative of three experiments. Statistical analysis was implemented by using one-way analysis of variance with Sidak's test. **means P＜0.01, **** means P < 0.001. Source data are provided as a Source Data file.

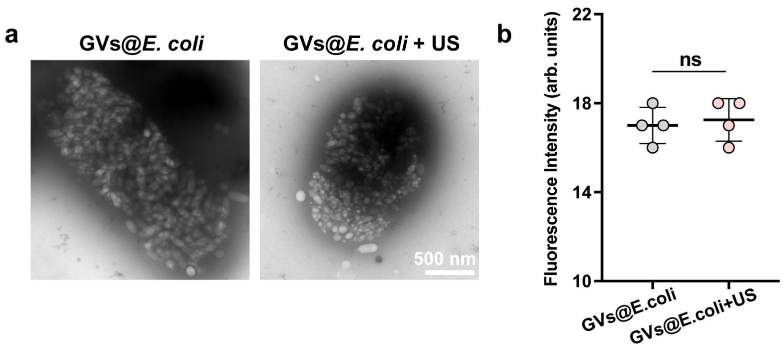

Supplementary Fig. 4 | Structural and chemical changes after acoustic irradiation of GVs@*E.coli*.
a Transmission electron microscope image of GVs@*E.coli* with or without ultrasound. Images were representative of three experiments. **b** The production of reactive oxygen species of GVs@*E.coli* with or without ultrasound, n = 4 biologically independent samples per group. Data were presented as mean ± s.d. Statistical analysis was implemented by using t-test. Source data are provided as a Source Data file.

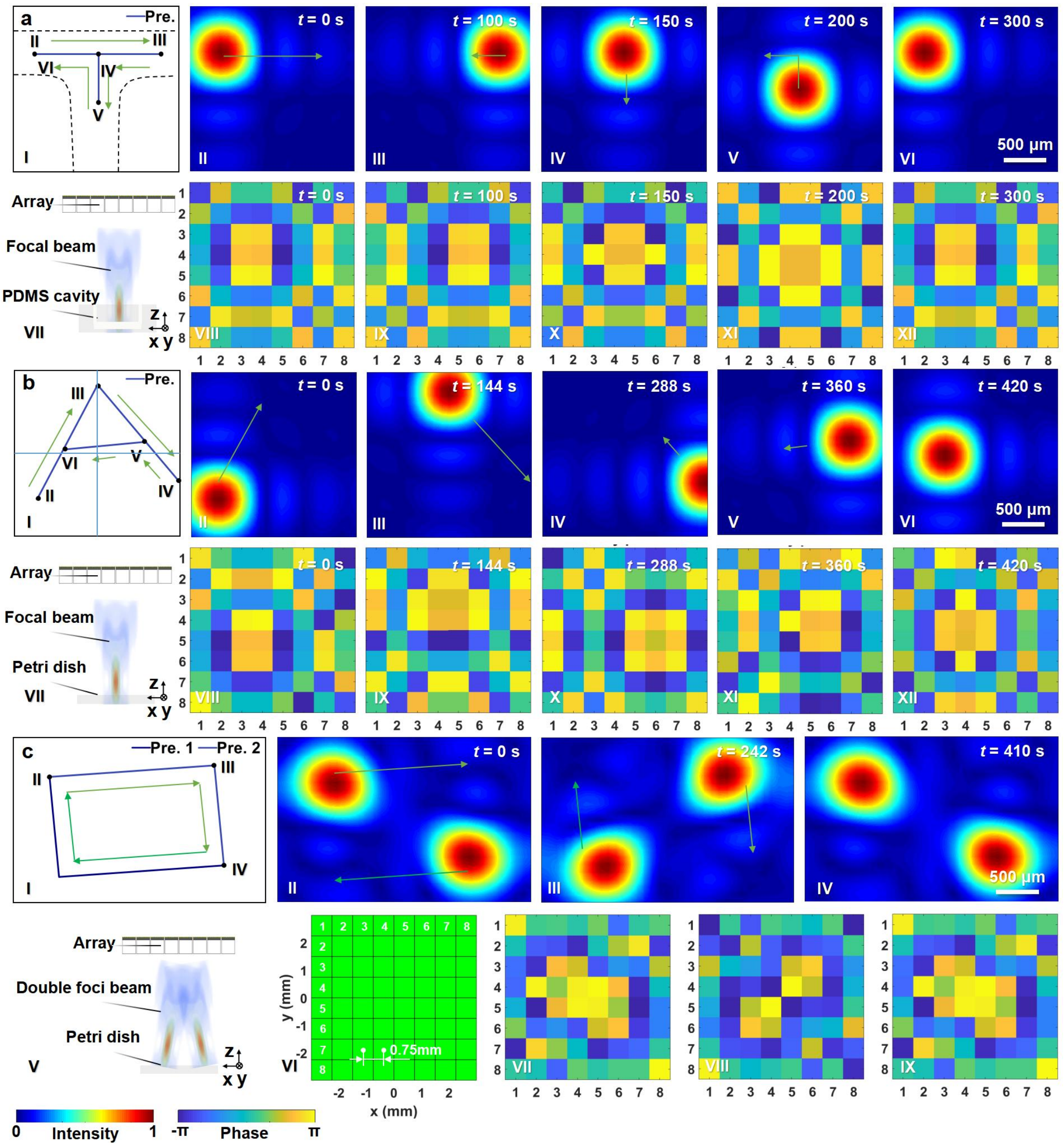

Supplementary Fig. 5 | Electronic steering of the focal acoustic beams. a Acoustic field setup for manipulating the GVs@*E. coli* cluster selectively through the fork in a T-shaped PDMS cavity. **b** Acoustic field setup for manipulating the GVs@*E. coli* cluster along an A-shaped trajectory under boundary-free conditions. I in **a** and **b**: Preset locomotion trajectory of cluster. II–VI in **a** and **b**: Time-lapse acoustic field sequences of the locations of the manipulated GVs@*E. coli* cluster. VII in **a** and **b**: Schematic diagram of manipulation experiment settings. VIII–XII in **a** and **b**: Time-lapse array phase sequences corresponding to the acoustic fields in II–VI. The green arrows indicate the translation directions. **c** Acoustic field setup for manipulating two GVs@*E. coli* clusters simultaneously. I: Preset locomotion trajectories of the two clusters. II–IV: Time-lapse multi-focus acoustic field sequences of the locations of the manipulated clusters. V: Schematic diagram of manipulation experiment settings. VI: Schematic diagram of array element distribution. VII–IX: Time-lapse array phase sequences corresponding to the acoustic fields in II–IV. The dark and light green arrows represent the different directions of movement of the two clusters, respectively.

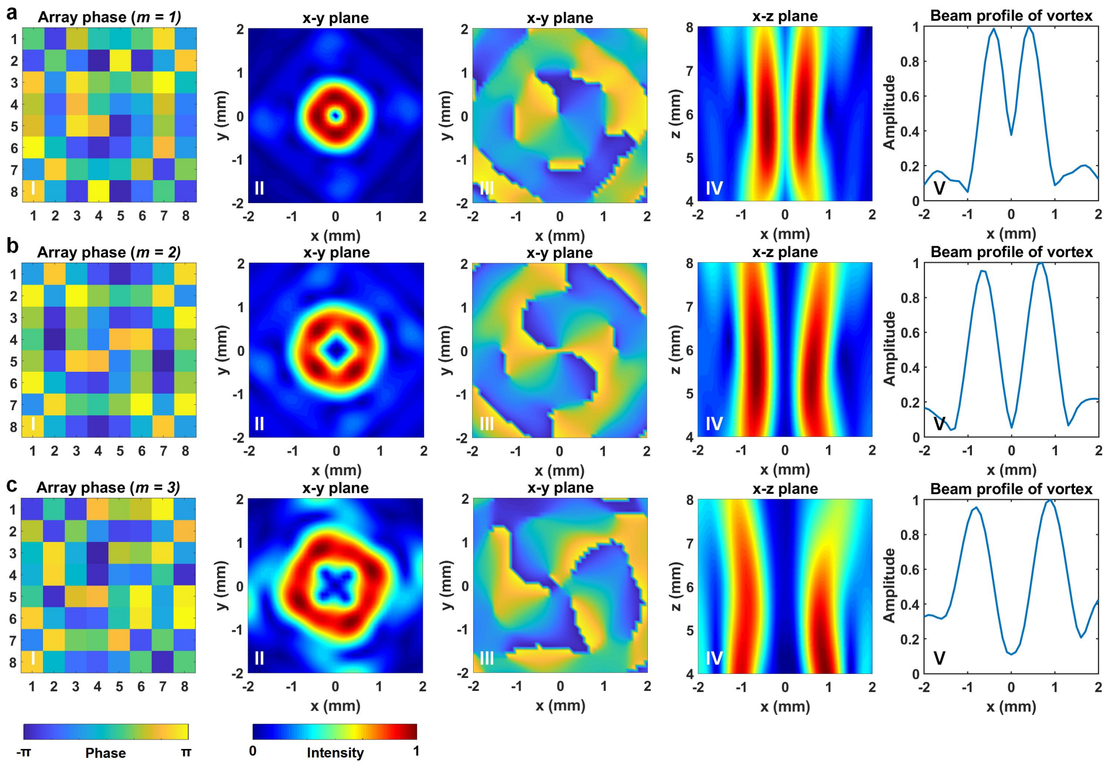

Supplementary Fig. 6 | Acoustic fields of vortices. a Characterization of vortex acoustic field (*m* = 1). **b** Characterization of vortex acoustic field (*m* = 2). **c** Characterization of vortex acoustic field (*m* = 3). I, II, III, IV, and V in **a**, **b**, and **c** are the array phase, x-y plane intensity (focal depth = 6 mm), x-y plane phase (focal depth = 6 mm), x-z plane intensity (y = 0), and beam profile (focal depth = 6 mm, and y = 0) of the vortex acoustic field when *m* = 1, 2, and 3, respectively.

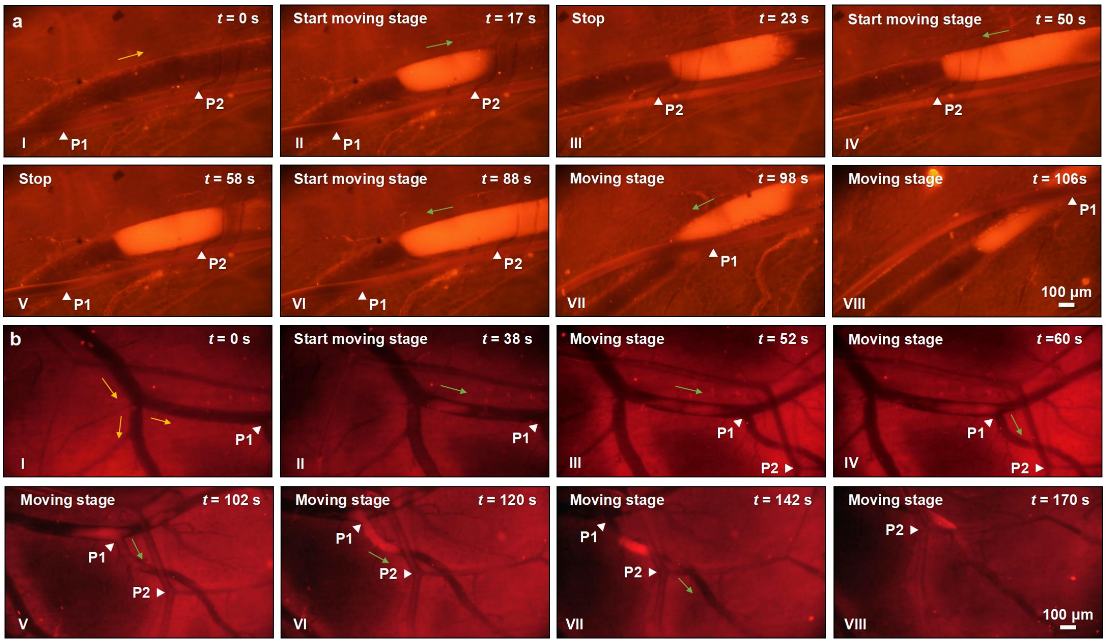

Supplementary Fig. 7 | *In-vivo* acoustic manipulation of GVs@*E. coli* cluster by moving the microscope stage. a Trapping the GVs@*E. coli* cluster to move back and forth along the blood vessel by moving the stage, and making the cluster stop movement at specific node positions. I–VIII: Time-lapse microscopic fluorescence image sequences of the location of the trapped GVs@*E. coli* cluster, where the cluster stopped at positions III and V. **b** Trapping the GVs@*E. coli* cluster selectively through the fork of the vessels by moving the stage. I–VIII: Time-lapse microscopic fluorescence image sequences of the location of the trapped GVs@*E. coli* cluster. Yellow arrows in **a** and **b** indicate blood flow direction. Two triangles P1 and P2 in **a** and **b** are given as reference points to show the location change of the vessel, whereas the green arrows indicate the direction of cluster movement.

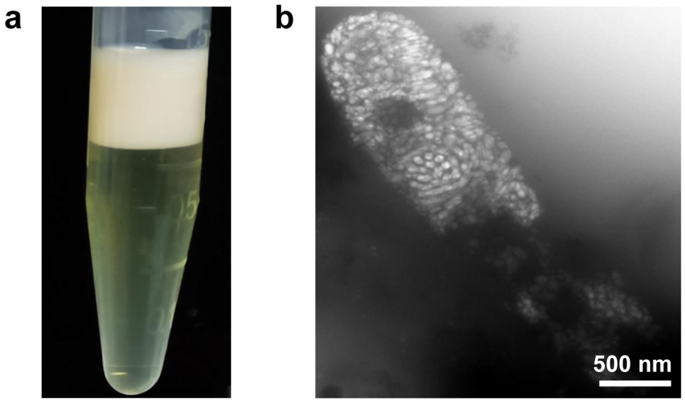

Supplementary Fig. 8 | GVs expression of GVs@YB1. a GVs@YB1 were collected into PBS solution and GVs@YB1 floated to the top of the solution after standing for 24 h (white). **b** Transmission electron microscope image of GVs@YB1. GVs were filled with the cytoplasm. Images were representative of three experiments.

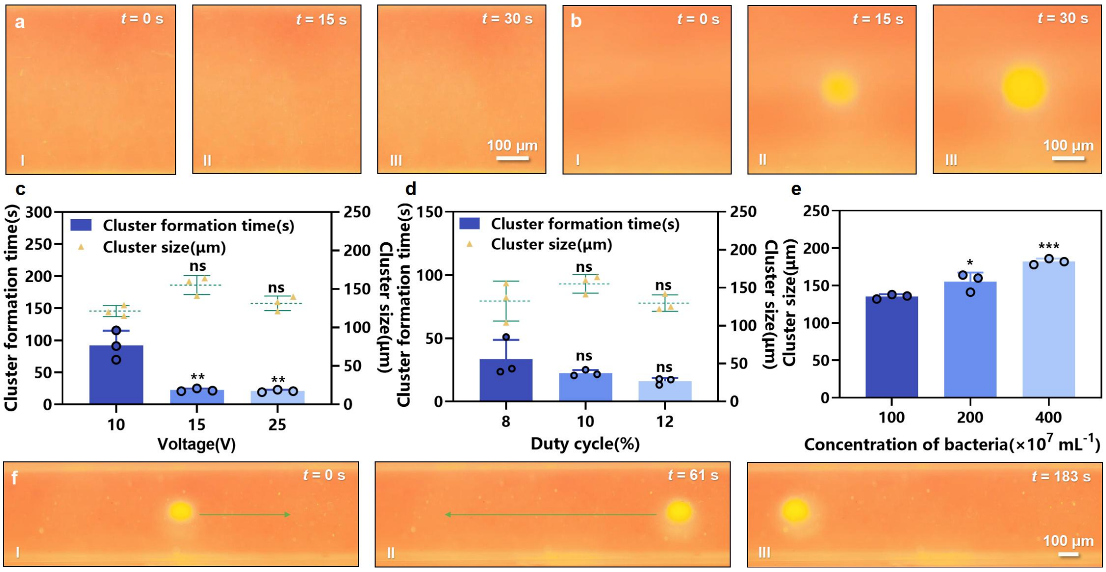

Supplementary Fig. 9 | Acoustic trapping and manipulation of GVs@YB1. Microscopic fluorescence photographs of **a** control YB1 and **b** GVs@YB1 in silicone tube being exposed to ultrasound (focal beam) for 30 s, respectively. Only GVs@YB1 can aggregate at the focal beam centre and form clusters under the dominance of the ARF. Images were representative of three experiments. Bacterial cluster formation times (100 μm cluster diameter) and cluster sizes (after ultrasound had been turned on for 2 min) of the GVs@YB1 under different **c** input voltages and **d** duty cycles. ** means P = 0.0017 (15 V group), P=0.0015 (25 V group) compared to the 10 V group in **c**. **e** Bacterial cluster size of the GVs@YB1 with various concentrations under the same ultrasound conditions. * means P = 0.0457 (200 × 10^7 cell mL^{-1} group) and *** means P = 0.0007 (400 × 10^7 cell mL^{-1} group) compared to the 100 × 10^7 cell mL^{-1} group in **e**. **f** Manipulating the GVs@YB1 cluster along a straight line within the range of ±0.6 mm by electronically steering the focal beams. Data in **c**, **d**, and **e** are presented as mean ± s.d. (n = 3 biologically independent samples per group). Statistical analysis was multiple comparisons by using one-way analysis of variance with Tukey's test. Source data are provided as a Source Data file.

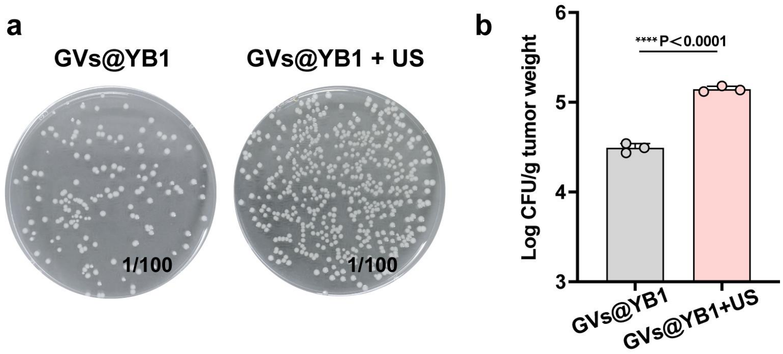

Supplementary Fig. 10 | Acoustic aggregation of GVs@YB1 in tumour (day 1). a The number of GVs@YB1 in the tumour of tumour-bearing mice with or without ultrasonic irradiation, at day 1 after intravenous administration of $5×10^7$ CFU GVs@YB1. **b** The Quantification of (a), in the day 1, the fold-difference between GVs@YB1 + US and GVs@YB1 reached 4.5, n = 3 biologically independent samples per group. Data were presented as mean ± s.d. Statistical analysis was implemented by using t-test. **** means P < 0.0001. Source data are provided as a Source Data file.

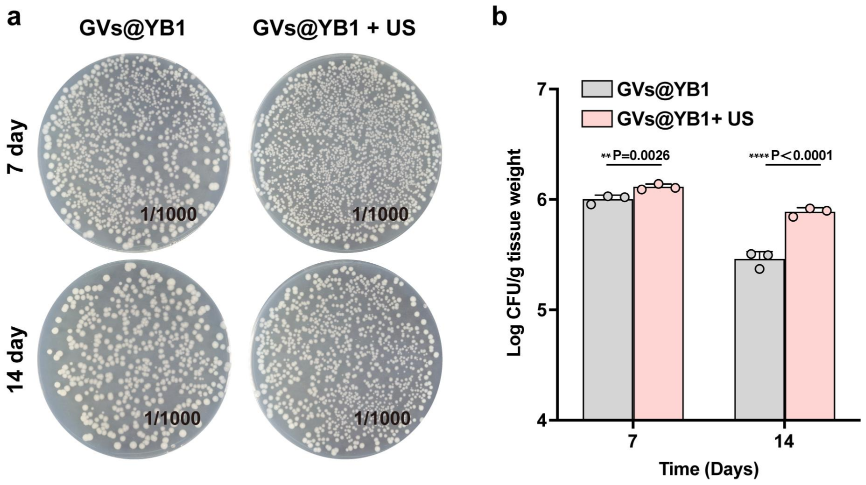

Supplementary Fig. 11 | Acoustic aggregation of GVs@YB1 in tumour (day 7 and day 14). a The number of GVs@YB1 in the tumour of tumour-bearing mice with or without ultrasonic irradiation, at day 7 and day 14 after intravenous administration of $5×10^7$ CFU GVs@YB1. **b** Quantification of **a**, in the day 7, the fold-difference between GVs@YB1 + US and GVs@YB1 reached 1.29, and 2.67 in day 14, n = 3 biologically independent samples per group. Data were presented as mean ± s.d. Statistical analysis was implemented by using two-way analysis with Sidak's test. ** means P < 0.01, and **** means P < 0.0001. Source data are provided as a Source Data file.
