# Supplementary material for: In-vivo programmable acoustic manipulation of genetically engineered bacteria
Source: Nat Commun. 2023 Jun 6;14:3297. doi: 10.1038/s41467-023-38814-w (PMC10244463; doi:10.1038/s41467-023-38814-w)
Supplement: Supplementary file 3 — Description of additional supplementary files [file 41467_2023_38814_MOESM3_ESM.pdf]

<u>Description of additional supplementary files</u>

Supplementary Movie 1 : shows the in-vitro acoustic manipulation of genetically engineered bacteria. When control E. coli and GVs@E. coli both being exposed to ultrasound (focal beam), only GVs@E. coli can aggregate at the focal beam centre and form clusters under the dominance of the ARF. Subsequently, the GVs@E. coli cluster can be manipulated alone a programmable trajectory by electronically steered focal beams, such as selectively through the fork in a T-shaped PDMS cavity, or along the A-shaped trajectory under boundary-free conditions. Two GVs@E. coli clusters can also be manipulated simultaneously along the boundary of the rectangle. And the transformation of the vortex acoustic field patterns (where the topological charge changed from m = 1 to m = 3) can be displayed by using GVs@E. coli.

Supplementary Movie 2 : shows the in-vivo acoustic manipulation of genetically engineered bacteria. The acoustic trapping of GVs@E. coli and control E. coli has been compared in superficial blood vessels on the backs of mice, respectively. Only GVs@E. coli can be trapped at the focal beam centre and form clusters in the vessels. Acoustic trapping of GVs@E. coli in blood vessels of different diameters has also been displayed (110-, 130-, 170-, and 200-μm-diameter vessels). Subsequently, the GVs@E. coli cluster can be manipulated in vivo by electronically steered focal beams. Firstly, the GVs@E. coli cluster can be manipulated to move back and forth along the blood vessel, while the cluster can stop moving for 30 s at specific node positions. Secondly, the GVs@E. coli cluster can be driven selectively through the fork of the vessels. Thirdly, two clusters can also be trapped simultaneously in the same blood vessel and manipulated to move closer or farther away from each other. Moreover, by moving the microscope stage, the trapped GVs@E. coli cluster can also be driven to move along the blood vessel.
